# A Polymer Visualization System with Accurate Heating and Cooling Control and High-Speed Imaging

**DOI:** 10.3390/ijms16059196

**Published:** 2015-04-23

**Authors:** Anson Wong, Yanting Guo, Chul B. Park, Nan Q. Zhou

**Affiliations:** 1 Department of Mechanical and Industrial Engineering, University of Toronto, Toronto, ON M5S 3G8, Canada; E-Mails: awong@mie.utoronto.ca (A.W.); guoyant@yorku.ca (Y.G.); 2 National Engineering Research Center of Novel Equipment for Polymer Processing, the Key Laboratory of Polymer Processing Engineering, Ministry of Education of China, South China University of Technology, Guangzhou 510641, China; E-Mail: nqzhou@scut.edu.cn

**Keywords:** foam, polypropylene, crystallization, high-pressure differential scanning calorimetry, foaming visualization

## Abstract

A visualization system to observe crystal and bubble formation in polymers under high temperature and pressure has been developed. Using this system, polymer can be subjected to a programmable thermal treatment to simulate the process in high pressure differential scanning calorimetry (HPDSC). With a high-temperature/high-pressure view-cell unit, this system enables *in situ* observation of crystal formation in semi-crystalline polymers to complement thermal analyses with HPDSC. The high-speed recording capability of the camera not only allows detailed recording of crystal formation, it also enables *in situ* capture of plastic foaming processes with a high temporal resolution. To demonstrate the system’s capability, crystal formation and foaming processes of polypropylene/carbon dioxide systems were examined. It was observed that crystals nucleated and grew into spherulites, and they grew at faster rates as temperature decreased. This observation agrees with the crystallinity measurement obtained with the HPDSC. Cell nucleation first occurred at crystals’ boundaries due to CO_2_ exclusion from crystal growth fronts. Subsequently, cells were nucleated around the existing ones due to tensile stresses generated in the constrained amorphous regions between networks of crystals.

## 1. Introduction

This article presents an expanded research study based a conference paper published in the Society of Plastics Engineers Annual Technical Conference 2012 [1].

High-pressure differential scanning calorimetry (HPDSC) is a widely used technology to determine the melting and crystallization kinetics of semi-crystalline polymers in the presence of dissolved gas at a high temperature and pressure [2,3,4,5]. However, many HPDSC systems have limited operating pressure (e.g., <15 MPa) when a high temperature (e.g., >150 °C) is used. Moreover, this thermal analysis could not provide direct information on the crystal population density and sizes. In this context, Mezghani and Philips [6] have incorporated a miniature high-temperature/high-pressure view-cell onto a polarized optical microscope (POM) to observe crystal formation of semi-crystalline polymers under a high temperature (200 °C) and pressure (200 MPa). The scientific knowledge of crystallization and melting behaviors generated from the thermal analysis and optical microscopy is imperative to the advancement of polymer science.

In addition, the understanding of crystallization/melting is critical to the development of plastic foaming science and technologies. This is because the formation of crystals has significant effects on cell nucleation [7,8,9,10,11,12,13,14,15,16,17,18], growth [15,18,19,20,21,22,23,24], and stabilization processes [13,16,24,25,26,27,28,29] that determines the final cellular structure (e.g., bubble size distribution and density, porosity, and volume expansion ratio), and hence the mechanical, thermal, acoustical, electrical and optical properties of the foams. A number of theories have been proposed to explain crystal nucleation, growth, and crystal-induced cell nucleation behavior (e.g., crystals as heterogeneous nucleating agents [14], crystals growth-induced gas exclusion leading to supersaturation of gas in the surrounding regions [30]). In addition, it was claimed that the foaming action further increases the crystallinity through biaxial orientation [31]. However, in order to enhance our fundamental understanding in this subject matter, further investigation is needed to confirm the validity of these theories, and/or to identify other possible crystal-induced foaming mechanisms.

One method to advance our understanding in this subject is to conduct *in situ* observation of plastic foaming processes. In 1978, Han *et al.* [32] and Villamizar [33] conducted pioneering research on *in situ* observations of plastic foaming processes through transparent slit dies and transparent mold cavities, which sparked numerous investigation in in-line foaming observation in extrusion foaming and injection foam molding processes [34,35,36,37,38,39]. In addition, Han *et al.* [40,41,42] examined a light-scattering method to detect the onset of cell nucleation by monitoring the electrical signal from a photomultiplier that collected scattered-light caused by phase separation in plastic melt. Meanwhile, Tatibouët and Gendron developed an in-line foaming detection technique based on ultrasonic measurement, where phase separation due to foaming was detected by sound attenuation and velocity [43,44]. These research studies have provided a significant insight on the plastic foaming behaviors within processing equipment. On the other hand, the bubble nucleation and growth phenomena in a continuous flow of plastic-gas solutions are highly complex, and their coupled thermodynamics, multi-phase fluid dynamics, and rheological processes are difficult to thoroughly understand.

In this context, previous researchers have developed batch view-cell systems to capture plastic foaming *in situ* with optical imaging under static [34,45,46,47] and controlled dynamic conditions [48,49,50,51], which enable in-depth investigations on various material compositions (e.g., base polymer, blowing agents, cell nucleating agents, *etc.*), experimental parameters (e.g., temperature, pressure, pressure drop rate, extensional strain, shear strain, *etc.*). These systems incorporated a heating unit and a temperature sensor to control the system temperature, but many of them do not have an active cooling mechanism. Consequently, they could not induce a rapid cooling and control the cooling rate accurately. This poses difficulty to control the crystal formation processes, which are strongly dependent on the thermal history of the polymer-gas mixtures. The high-pressure POM system developed by Mezghani and Philips [6] is capable to observe crystallization under high temperature and pressure. However, it does not have a rapid depressurization element to control the pressure drop rate, which is critical for foaming processes. In addition, it does not have the high-speed imaging capability. The high-speed imaging capability is necessary to capture plastic foaming processes *in situ* since cell nucleation and growth processes could be completed within tens or hundreds milliseconds, especially when a high gas concentration and/or a high pressure drop rate are used.

In this context, this paper presents the development of a static foaming visualization system with a high-speed camera and active heating and cooling controls. This system enables *in situ* observation of crystal formations under a high temperature and pressure, which complements HPDSC analysis to achieve in-depth understanding of crystallization and melting behaviors of semi-crystalline polymers under typical foaming conditions. At the same time, it allows *in situ* observation of the subsequent plastic foaming processes induced by a rapid depressurization. Combining these two capabilities, this system could be effectively used to examine crystal formation and their effects on plastic foaming behavior of semi-crystalline polymers. In order to verify the capability of this system, the crystal formation and foaming processes of a linear PP and a PP-ethylene copolymer blown with high-pressure CO_2_ has been observed *in situ*. Through this investigation, the effects of crystals on the cell nucleation and growth behavior have also been identified. In addition, thermal analysis of these polymers conducted at the same thermal cycles has also been evaluated with high-pressure differential scanning calorimetry (HPDSC) to compare with the crystal formation data generated with the foaming visualization system.

## 2. Results and Discussion

### 2.1. System Development

**Figure 1:**
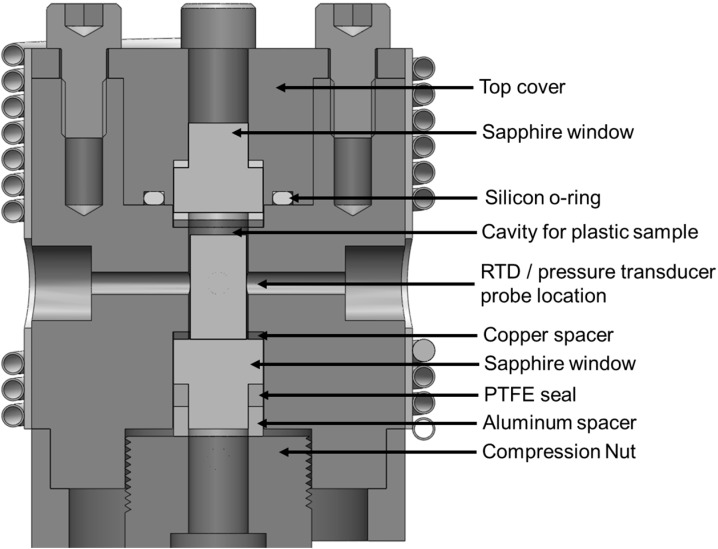
Detailed chamber design for the polymer visualization system.

The accurate heating/cooling control is essential to studies where the thermal history of a plastic sample impact its foaming behavior, notably the foaming of semi-crystalline polymers at temperatures where crystallization occurs, such as in many bead foaming processes. Therefore, based on the foaming visualization system developed by Guo *et al.* [46], a new view-cell system has been developed that incorporates a water-cooling system. The new view-cell was constructed with a smaller chamber body than the existing one to achieve a faster heating/cooling response. The chamber body was made with Inconel due to its high resistance to corrosion. The contact area between the chamber and its supporting stand has also been minimized to suppress heat dissipation. It also adopted a cylindrical uni-body design in place of the multi-layered existing chamber. This new design eliminated the need to realign the chamber layers during sample loading and replacement of high-pressure gas-sealing gasket in between experimental runs. A sapphire window was installed at the bottom surface of the chamber to provide transmissive lighting, while another sapphire window was installed on the top cover for bright field observation. The sapphire-to-metal sealing mechanism for both the top and bottom sapphire windows has been designed based on our previous works [48,49], which was developed based on O.M. Suleimenov’s design [52]. The basic concept behind the sealing mechanism is the Bridgman’s unsupported area principle [53], which involves generating a high pressure on the sealing element (e.g., an o-ring) from the internal pressure of the chamber. The increased pressure deforms the sealing element, which then penetrates the surrounding gaps and improves the seal’s performance. To utilize this principle, a mushroom-shaped sapphire window has been used to guide a sealing o-ring (see Figure 1 for details). A compression nut was installed to provide the clamping force required for the initial seal. The area of the sapphire window under pressure was designed to be larger than the facial area of the o-ring, thus the pressure exerted on the o-ring would be higher than the internal pressure. The mushroom-shaped sapphire window prevented the o-ring from deforming excessively towards the center under pressure that could cause damages to the o-ring. To avoid damaging the sapphire window against the chamber body during the initial seal, a copper ring was sandwiched in between the window and the chamber. This sealing mechanism was superior to the previous design where an o-ring was sandwiched between the chamber body and the sapphire window. This is because the previous design relied on an external clamping force to deform the o-ring to form a seal; the external clamping force must also be uniform along the sealing surface to avoid damages to the o-ring and/or the sapphire window. In addition, as pressure increased, the external clamping force required to prevent leakage also increased. On the other hand, the new design only required a moderate clamping force for the initial seal, and the clamping force on the o-ring increased as the internal pressure increased. Therefore, this sealing design was more robust than the previous design. The material of the sealing o-ring has been chosen to be PTFE with 25% glass-fiber due to PTFE’s inertness and high service temperature limit (*i.e.*, 260 °C), and the high mechanical strength of glass-fiber that prevented excessive deformation of the o-ring even at high temperatures and pressures. A resistive thermal detector (RTD) (PT110, Omega Engineering, Stamford, CT, USA) and a pressure transducer (PT160, Dynisco, Franklin, MA, USA) have also been installed to measure the gas temperature and pressure inside the chamber.

This sealing mechanism has been used for both the top and bottom sapphire windows. At the bottom side, a threaded compression nut was used to clamp the sapphire window and sealing components together to provide an initial seal. The compression nut also secured an optic fiber that was connected to a halogen lamp as a transmissive light source. The design of the top compression nut was different from the bottom one such that the top sapphire window and the sealing o-ring were housed within the compression nut. This design allows for easier access to load and remove a plastic sample. However, this design also generated an additional leakage path between the top compression nut and the chamber body. Therefore, it has been sealed with a silicon-based o-ring installed on a slot on the compression nut. Four M6 bolts have been used to provide the clamping force for the top compression nut, instead of a threaded nut design, to avoid potential damages of threads from the frequent open and close operation of the top nut for sampling loading and removal. The top nut has a cylindrical lead-in that mates with the chamber body to a close-tolerance slide-fit, hence proper alignment between the top nut and the chamber would be guaranteed. This foaming chamber design has been tested to maintain good sealing performance after repeated uses without the need to replace any of the sealing o-rings on a frequent basis. For example, good sealing was achieved at 20 MPa at 240 °C, which is sufficient for conducting research on a wide range of plastic foaming processes. Additional tests will be conducted in the future to determine the maximum operating temperature and pressures of this system.

**Figure 2:**
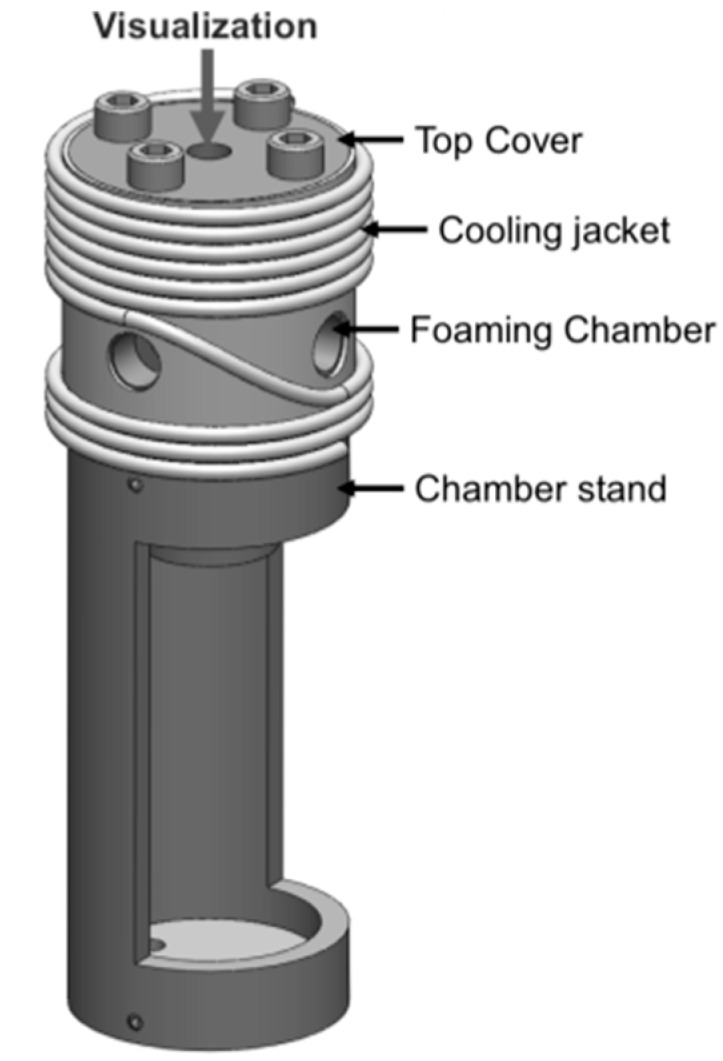
Overall chamber design of the polymer visualization system.

**Figure 3:**
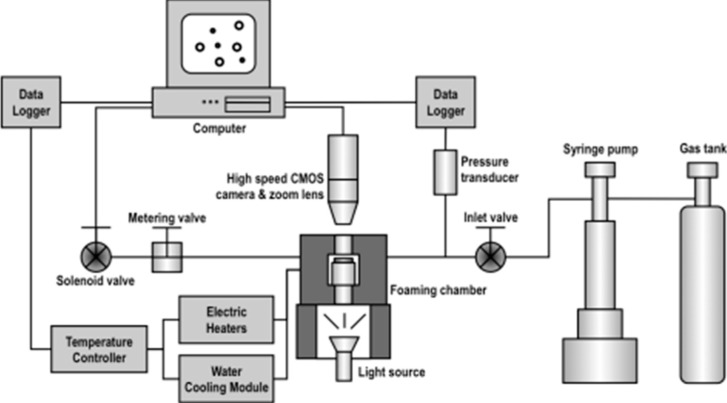
Polymer visualization system with accurate heating/cooling program control.

The accurate heating and cooling function was achieved with two electric cartridge heaters and a water-cooling module, respectively, and they were controlled by a single temperature controller (CN7833, Omega Engineering, Stamford, CT, USA). The cooling module consisted of a customized cooling jacket with surrounding copper tubes soldered to its surface. The tubes circulated cool water (maintained at 20 °C) that was supplied by a water line to achieve the cooling function. The water flow was controlled via the opening of a solenoid valve that was operated by the temperature controller. The cooling jacket was installed onto the chamber’s cylindrical surface. Thermal paste has been added to their contact area to ensure effective heat transfer Figures 2 and Figure 3 show the design of the foaming chamber with cooling jacket and the overall foaming visualization system, respectively. The temperature of the system was recorded into a computer program (CN7-A, Omega Engineering, Stamford, CT, USA) in real-time.

**Figure 4:**
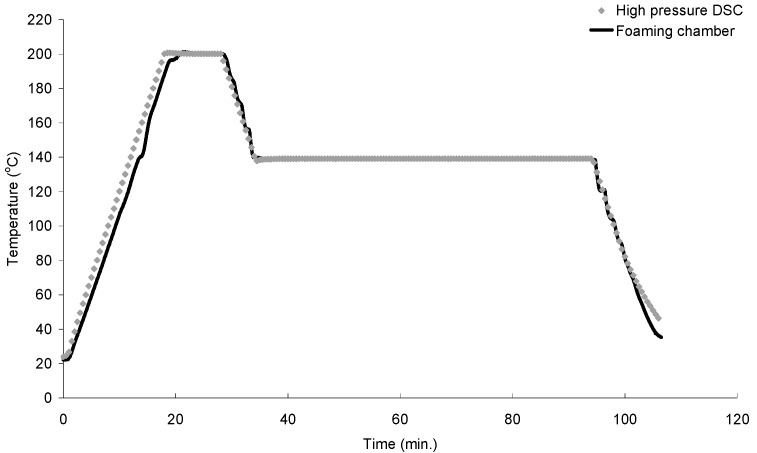
Temperature profile in the chamber *vs.* high pressure differential scanning calorimetry (HPDSC) with *T_iso_* = 139 °C.

To demonstrate the heating and cooling capability of the system, the temperature controller has been programmed to achieve a temperature profile for the foaming chamber, pressurized to 6 MPa with CO_2_, as follows: (1) heat from 20 to 200 °C at 10 °C/min; (2) maintain at 200 °C for 10 min; (3) cool to 139 °C at 10 °C/min; (4) maintain at 139 °C for 60 min; and (5) cool to 20 °C at 10 °C/min. Initially, the auto-tuning feature has been used to tune the PID parameters for the temperature control. However, during the cooling stage from 200 to 139 °C, the actual temperature overshot a few degrees below the set isothermal temperature (*T_iso_*). Since isothermal crystallization phenomenon in semi-crystalline polymers is strongly dependent on temperature, an overshoot in temperature decrease might accelerate the nucleation of crystals, which causes inconsistency in experimental results. To overcome this issue, the proportional, integral and differential parameters of the controller had been tuned manually to eliminate the overshoot while maintaining the specified heating/cooling rate and the holding temperature at each stage. The temperature readings are shown in Figure 4. The same temperature profile obtained with a high-pressure differential scanning calorimeter (HPDSC) (DSC 204 HP, NETZSCH, Selb, Bavaria, Germany) has also been included for comparison, which shows that the two profiles are very similar. This demonstrated the accurate heating/cooling capability of the foaming system. To be specific, for both systems, the maximum temperature overshot at the beginning of the isothermal temperature stage was very small (less than 1 °C) and the systems quickly stabilized at *T_iso_* (see Figure 4). Therefore, the impact of temperature fluctuation on the isothermal crystallization behavior of semi-crystalline polymers (e.g., polypropylene) should be negligible.

In order to verify the capability of this system and to elucidate the effects of crystals on the cell nucleation and growth behavior, the crystal formation and foaming processes of a linear polypropylene (PP) and a PP-ethylene copolymer blown with high pressure CO_2_ has been observed *in situ* and the results are described in the following sections. In addition, thermal analysis of these polymers conducted at the same thermal cycles has also been evaluated with HPDSC to compare with the crystal formation data generated with the foaming visualization system. Previous studies reported that scan speed (*i.e.*, the heating/cooling rate during a ramp) of DSC analysis had significant effects on the crystallization behaviors of PP: (i) the melting temperature and heat of fusion increased as the heating rate increased [54]; (ii) the crystallization temperature and heat of crystallization decreased with increasing cooling rate [55]; and (iii) under a higher cooling rate, crystallization started earlier and lasted for a shorter period of time, and the final degree of crystallinity was lower [56]. On the other hand, the goal of this study was to demonstrate the capability of the crystallization/foaming visualization system and that in-depth understanding of crystallization behavior of semi-crystalline polymers under typical foaming conditions could be achieved using this visualization system in conjunction with HPDSC. Therefore, the cooling rate effect was not examined in this study, and the heating/cooling rate was kept constant at 10 °C for both the visualization and HPDSC experiments.

### 2.2. Measurement of Isothermal Crystallization with High-Pressure Differential Scanning Calorimetry

**Figure 5:**
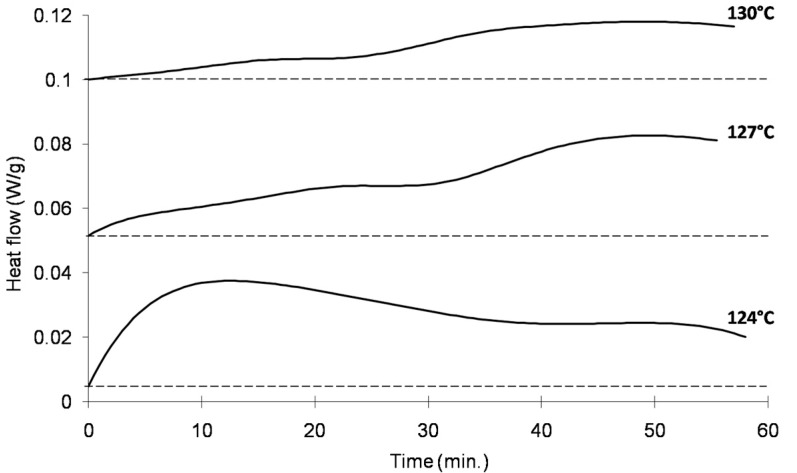

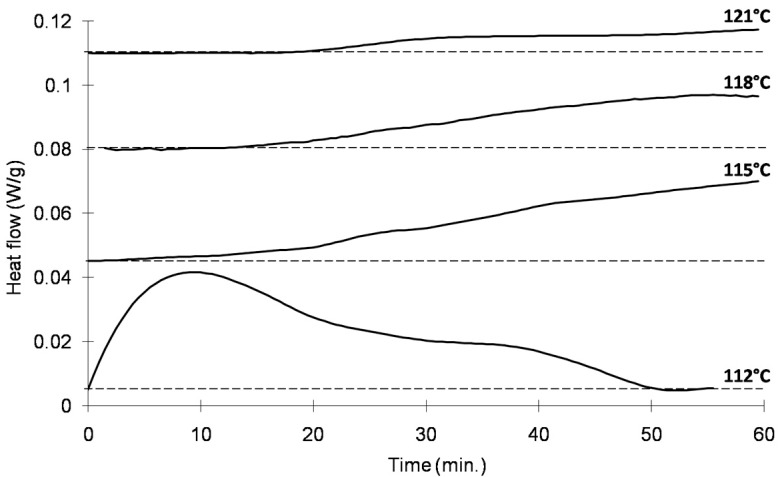
(**a**) Isothermal crystallization of the linear PP using high pressure DSC (*P_sat_* = 6 MPa); (**b**) Isothermal crystallization of the PP-ethylene copolymer using high pressure DSC (*P_sat_* = 6 MPa).

**Figure 6:**
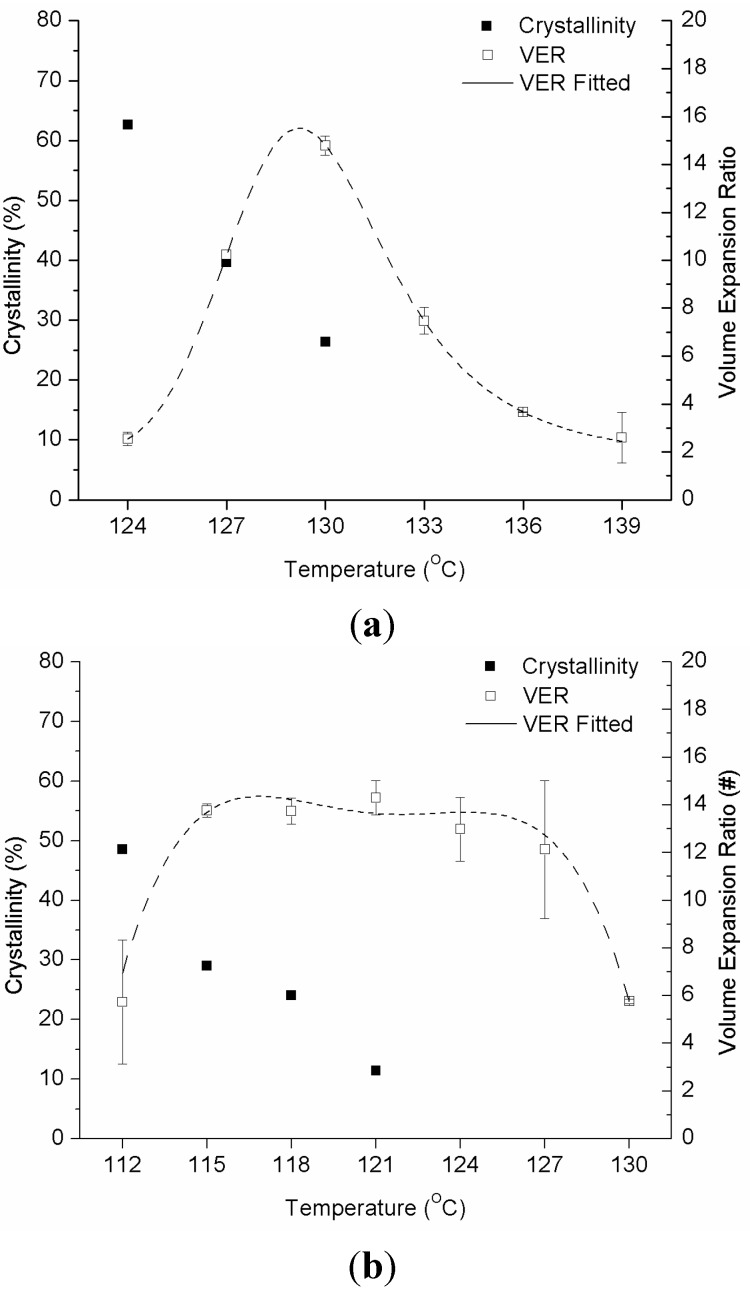
(**a**) Crystallinity and VER *vs.* *T_iso_* for the linear PP; (**b**) Crystallinity and VER *vs.* *T_iso_* for the PP-ethylene copolymer.

The high-pressure differential scanning calorimetry (HPDSC) results for the isothermal sections of a linear PP and PP-ethylene copolymer are shown in Figure 5a,b, respectively (refer to Figure 4 and Section 3.2 for details of the experimental method). The CO_2_ pressure (*P_sat_*) used in all experiments were 6 MPa. The *T_iso_* ranges used for the linear PP and PP-ethylene copolymer are 124 to 130 °C and 112 to 121 °C, respectively, at 3 °C intervals. For both materials, the crystallinity decreased as temperature increased. Figure 6a,b summarizes the crystallinity at the end of the 60-min isothermal process for all cases (*i.e.*, at the time when foaming would be induced by rapid depressurization for the foaming visualization experiments). Note that the volume expansion ratio (VER) of the foams generated from the subsequent foaming experiments is also included in these figures, which are explained in the following section.

### 2.3. In Situ Observation of Crystal Formation and Foaming Processes

The *T_sys_* profile in each *in situ* crystallization and foaming visualization experiment was the same as the HPDSC analysis up to the end of the 60 mins isothermal annealing stage, hence the crystallization analysis obtained with both systems were directly comparable. Afterward, a faster cooling rate was used in the foaming experiment in order to stabilize the foam structure quickly for analysis. For the foaming experiments, the *T_iso_* ranges used for the linear PP and PP-ethylene copolymer are 124 to 139 °C and 112 to 130 °C, respectively, at 3 °C intervals. These ranges covered those used in the HPDSC analysis, as well as higher *T_iso_* to study the foaming behavior where the occurrence of isothermal crystallization was not apparent in the HPDSC analysis. The *P_sat_* and the average pressure drop rate (−*dP_sys_/dt*|_avg_) were kept constant at 6 MPa and 2.1 MPa/s in all cases. Table 1 summarizes the experimental conditions. Each experiment was conducted three times to ensure that the results were repeatable.

**Table 1:** PP/CO_2_ experimental matrix.

Material	*T_iso_* [°C]	Material	*T_iso_* [°C]
Linear PP	124	PP-ethylene copolymer	112
	127		115
	130		118
	133		121
	136		124
	139		127
			130

*P_sat_* = 6 MPa and −*dP_sys_*/*dt*|_avg_ = 2.1 MPa/s.

**Figure 7:**
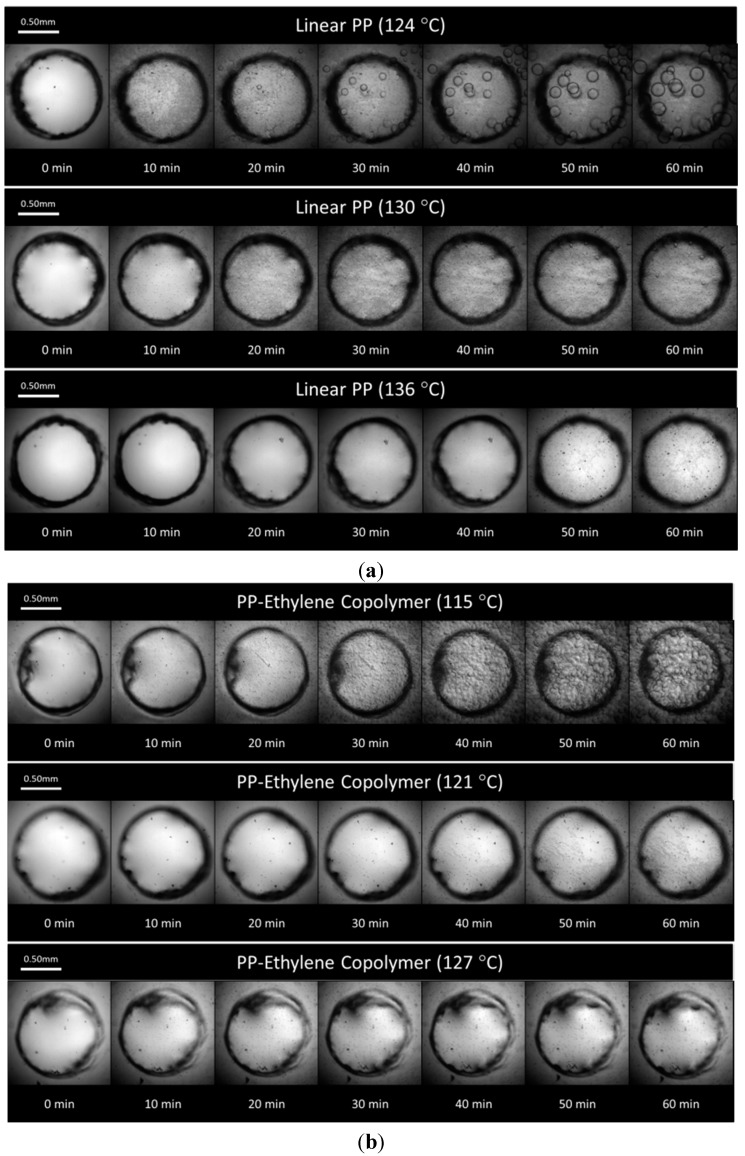
(**a**) Isothermal crystallization of the linear PP; (**b**) Isothermal crystallization of the PP-ethylene copolymer.

Using the crystallization/foaming visualization system, images of PP samples were taken at 1-min intervals during the 60 min isothermal stage to capture the crystallization behavior at the same conditions. Figure 7a,b shows snapshots of the crystallization behavior for the linear PP and PP-ethylene copolymer, respectively. It was observed that as the temperature decreased, the crystals’ density and growth rates were higher. In particular, for the linear PP, some crystals with sizes around 200 µm were observed at the end of the isothermal stage at 124 °C, while the average crystal sizes observed at the end of the isothermal stage at 136 °C case was only 15 µm (see Figure 7a). The crystal sizes for the PP-ethylene copolymer at low *T_iso_* was not measureable since the crystals interacted with each other and their boundaries were not distinct. However, the total area occupied by crystals was much higher than the high *T_iso_* cases as seen in Figure 7b. These observations agree with the decrease of crystallinity measured in HPDSC as the *T_iso_* increased. However, spherulites contain lamellar as well as amorphous regions between the lamellae [57]. Since these interlamellar amorphous regions could not be easily differentiated with the optical system, it is not possible to accurately determine the volume and hence the mass fraction of crystals with the foaming visualization system. Therefore, direct comparison between the HPDSC and visualization system in crystallinity *vs.* time is not possible. However, the images of crystal growth and interaction under high temperature and pressure still provide significant insights that could not be obtained with the HPDSC.

**Figure 8:**
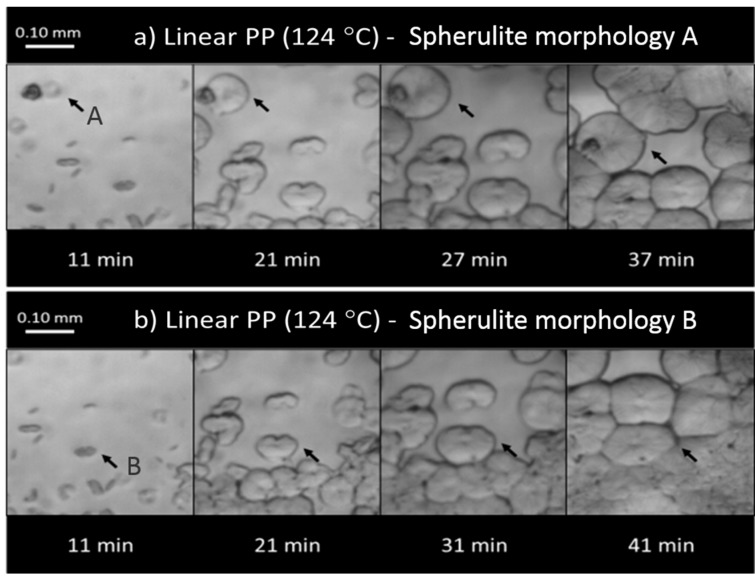
Spherulite growth behavior of the linear PP: (**a**) Spherulite morphology A; (**b**) Spherulite morphology B (the arrows indicate an example of the respective crystal growth behavior).

In particular, the crystal morphologies of linear PP were investigated and shown in Figure 8. It is well-known that there are three basic crystalline crystal structures of polypropylene (α, β, and γ) [58]. In this study, two kinds of spherulite morphologies were observed. One was the common spherulite (“A” in Figure 8a), in which the lamellar grew radially in all direction and demonstrated a spherical symmetry, while the other (“B” in Figure 8b) was not spherically symmetric and appeared as a sheaf-like structure. Through *in situ* observation during the isothermal stage, these two types of lamellar growth behavior were examined in detail (see Figure 8a,b). Spherulite A started from a central nucleus, and then grew radially in all directions to become a spherulite, with only small amount of cross-hatching. Subsequently, these spherulites continued to grow until they came in contact with adjacent crystals, which are called spherulite truncation (Figure 8a). Spherulite B developed into a sheaf-like lamellar structure initially, and then attained the spherical shape via continuous branching and fanning of the sheaf-like structure (*i.e.*, cross-hatching accompanied by a small amount of radial growth). The lamellae of these spherulites formed a crosshatched structure when they came in contact with adjacent ones without showing obvious boundaries ( Figure 8b). On the other hand, based on the available information, it is difficult to determine the crystal type (α, β, or γ).

**Figure 9:**
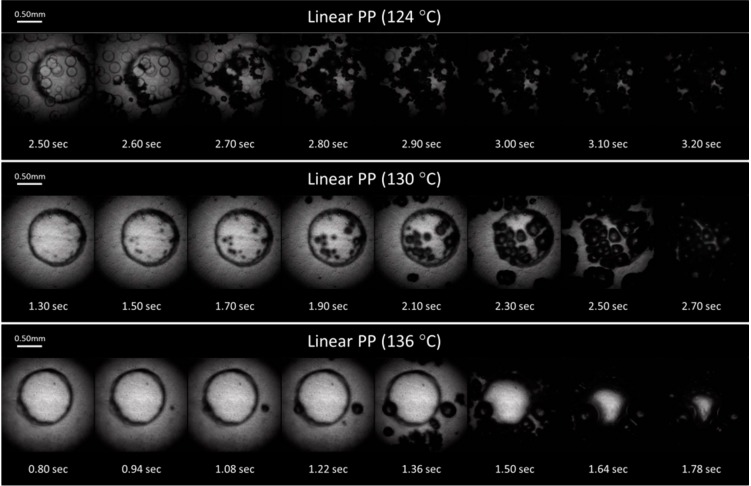

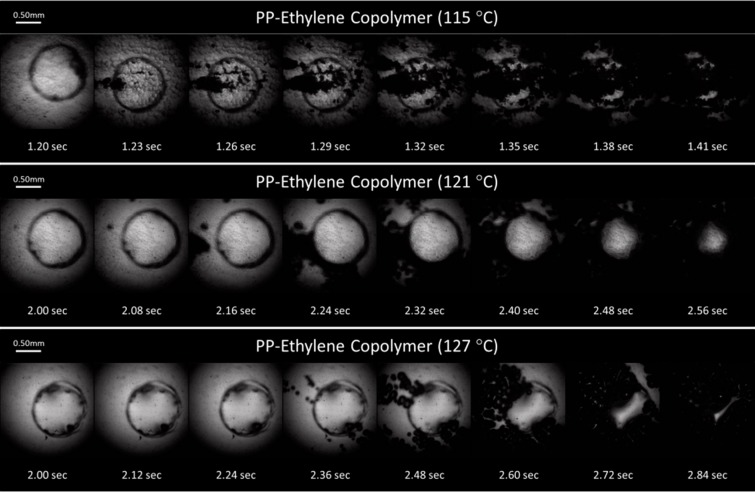
(**a**) Sample foaming visualization images of the linear PP; (**b**) Sample foaming visualization images of the PP-ethylene copolymer.

**Figure 10:**
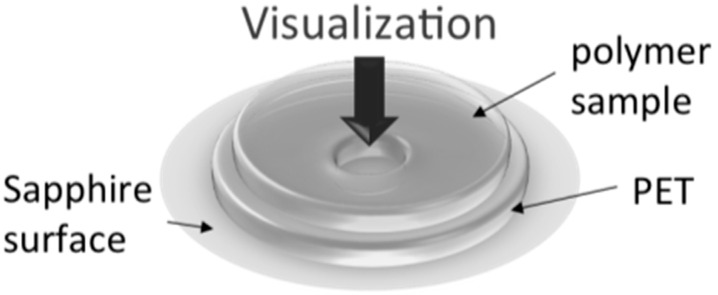
Foaming visualization at the suspended region.

Snapshots of the foaming videos from selected experiments of the linear PP and PP-ethylene copolymer are shown in Figure 9a,b, respectively. At a low foaming *T_iso_* (*i.e.*, *T_iso_* = ~8 °C above their individual crystallization temperature (*T_c_*)) and in the region where the plastic sample was suspended in the air (refer to Section 3.3 and Figure 10), the majority of the bubbles were nucleated around existing crystals (*T_iso_* = 124 °C for the linear PP and *T_iso_* = 115 °C for the PP-ethylene copolymer). They grew rapidly in the outward radial direction away from the crystal nuclei due to the high stiffness of the crystals. This crystal-induced cell nucleation could be explained by two main mechanisms.

The first mechanism was the exclusion of CO_2_ at the crystal growth front to the intercrystalline amorphous region as gas could not be dissolved in the crystalline phase. The expelled gas led to surrounding amorphous region becoming supersaturated and hence foaming occurred in this region. This CO_2_ exclusion effect was first proposed by Oda and Saito [30]; Koga and Saito [59] later confirmed this phenomenon as a viable foaming mechanism in their examination of the morphology of high-density polyethylene (HDPE) and poly(vinylidene fluoride) (PVDF) crystallized under high pressure CO_2_ with polarized optical microscopy, where they observed a fine-layered porous structure for both materials.

Another reason was the polymer chain networks formed around crystals. As crystallization occurred, polymer tended to shrink at the crystal sites. Consequently, the amorphous regions surrounding the crystals were constrained and tensile stresses were generated. When a bubble was nucleated in this constrained region, the growth of this bubble caused deformation to the surrounding polymer chains. Since these chains were constrained by the crystals, additional tensile stresses were generated in these regions. As shown in our previous works [13,48,49,60], tensile stresses could effectively induce cell nucleation (e.g., cell density increased by four orders of magnitude for a PS-talc composite as tensile stress was applied [60]). This is due to the decrease in system pressure (*P_sys_*) in local regions due to a tensile stress. The classical nucleation theory (CNT) [61,62] could be used to explain this phenomenon. According to the CNT, the critical radius (*R_cr_*) and the free energy (*W*) required for cell nucleation are given, respectively, as:

(1) $$R_{cr}=\frac{2{\text{γ}}_{lg}}{P_{bub,cr}-P_{sys}}$$

(2) $$W=\frac{16{\text{πγ}}_{lg}^{3}F}{3{\left(P_{bub,cr}-P_{sys}\right)}^{2}}$$

where γ*_lg_* is the surface tension at the liquid-gas interface, *P_bub,cr_* is the pressure of a bubble at the critical state, and *F* is the ratio of the volume of the nucleated bubble to the volume of a spherical bubble with the same radius of curvature, which is related to the surface geometry of the nucleating agent and the contact angle (θ*_c_*) between the polymer-gas mixture and the nucleating agent. For homogeneous nucleation, *F* equals 1. In order to account for the local pressure variation (*∆P_local_*), the *P_sys_* term in Equations (1) and (2) could be modified as [48,63]:

(3) $$R_{cr}=\frac{2{\text{γ}}_{lg}}{P_{bub,cr}-\left(P_{sys}+\Delta P_{local}\right)}$$

(4) $$W=\frac{16{\text{πγ}}_{lg}^{3}F}{3{\left(P_{bub,cr}-\left(P_{sys}+\Delta P_{local}\right)\right)}^{2}}$$

By considering the kinetics of cell nucleation [62], the cell nucleation rate (*J*) could be expressed as:

(5) $$J=N^{\frac{2}{3}}Q\sqrt{\frac{2{\text{γ}}_{lg}}{\text{π}mF}}exp\left(-\frac{W}{k_{B}T_{sys}}\right)=N^{\frac{2}{3}}Q\sqrt{\frac{2{\text{γ}}_{lg}}{\text{π}mF}}exp\left(-\frac{16{\text{πγ}}_{lg}^{3}F}{3k_{B}T_{sys}{\left(P_{bub,cr}-\left(P_{sys}+\Delta P_{local}\right)\right)}^{2}}\right)$$

where *N* is the number of gas molecules per unit volume of polymer; *Q* is the ratio of the surface area of the heterogeneously nucleated bubble to that of a spherical bubble with the same radius of curvature; *m* is the mass of a gas molecule; and *k_B_* is the Boltzmann’s Constant. In the presence of a tensile stress, Δ*P_local_* would be negative, thus the local *P_sys_* would be decreased. Consequently, the level of supersaturation, which is defined as the pressure difference between *P_bub,cr_* and the local *P_sys_*, would be increased, which led to reduction in *R_cr_* and *W*. Consequently, some existing microvoids that had radius greater than the decreased *R_cr_* would grow spontaneously to become nucleated cells. In addition, the cell nucleation rate (*J*) would also increase due to the increased level of supersaturation. This stress-induced nucleation mechanism explained why new bubbles were nucleated around existing bubbles and this created a chain effect that propagated into the surrounding regions quickly (refer to Figure 9a,b). These phenomena was similar to that observed by Leung *et al.* [63] in the foaming of PS-talc composites, where the presence of talc was believed to cause local stress fluctuations similar to that generated by crystals in this case. Importantly, it is noted that, while the exclusion of CO_2_ at crystals growth fronts was successful in explaining the initial foaming along the crystals’ boundary, it could not describe the bubble-growth induced nucleation phenomena observed; stress-induced nucleation is believed to be the dominant foaming mechanism in this study.

At higher *T_sys_*, these two foaming mechanisms became less apparent as the crystallinity and the viscosity of the polymer-gas mixtures decreased. As the crystallinity decreased, the exclusion effect of CO_2_ became less significant. In addition, the amorphous regions became less constrained and had lower viscosity, so the tensile stresses induced to polymer chains by bubble growth also decreased. Combined, these two phenomena caused reduction in nucleation rate, especially in the suspended region. In the region where PP was wetted on the PET surface, the PET-PP contact provided an additional constraint to polymer chains and hence they would be subjected to a higher amount of tensile stresses. This explained why the bubble-growth induced nucleation phenomena was still apparent in these regions but the propagation stopped at the suspended regions (refer to Figure 9b, *T_iso_* = 121 °C). At even higher temperatures, the bubble growth-induced nucleating phenomena were not observed due to the absence of crystals and the low viscosity of the polymer-gas mixture. However, bubbles were still nucleated on the PP-PET contact regions due to the heterogeneous nucleation effect of the PET. Meanwhile, no bubbles were nucleated in the suspended region, but the size of that region decreased as bubbles in the neighboring areas grew in sizes and caused deformation to this region.

Although it was observed that crystals induced cell nucleation, an excessive amount of large-sized crystals might hinder cell structure uniformity as cell growth around crystals would be restricted. The volume expansion ratio (VER) would also decrease due to the high viscosity of the polymer-gas mixture that restricted cell growth. An appropriate amount and sizes of crystals would induce cell nucleation uniformly, allow sufficient cell growth, as well as provide enough melt strength to prevent cell coalescence and collapse Figure 6a,b show the VER of the stabilized foams obtained in the foaming experiments *vs.* the isothermal/foaming temperature. The VER data was evaluated using the water-displacement technique based on ASTM D792-00. For both materials, a typical single-maximum-peak *vs.* temperature behavior [26] that showed a low VER due to limited cell growth in the presence of high crystallinity at low temperatures, as well as due to cell deterioration at high temperatures, was observed. The VER peak for the linear PP (maximum VER ≈ 15 times at *T_iso_* = 130 °C) was very narrow when compared to that of the PP-ethylene copolymer, which demonstrated the challenges in processing linear PP: it crystalizes quickly at low temperatures and exhibits a low melt strength at high temperatures. It was observed that a high crystallinity (~25%) was needed for the linear PP to achieve the maximum VER. This could be due to the low melt strength of linear PP, hence a larger amount of crystals were needed to increase the melt strength and to prevent significant gas loss. However, for processes with faster cooling (e.g., extrusion foaming and structural foam molding), foam stabilization occurs in shorter time, so a lower melt strength and hence crystallinity before foaming occurs would be sufficient to prevent cell deterioration. Therefore, the *T_iso_* at which maximum VER occurs is expected to be higher in these cases. Meanwhile, for the PP-ethylene copolymer, the volume expansion ratio maintained at the highest level (maximum VER ≈ 13 times) even when the crystallinity dropped below 10%. This was due to the inclusion of the ethylene chains in the PP-ethylene copolymer, which exhibited higher extendibility that prevented rupture of cell walls and hence foam shrinkage. Therefore, a smaller amount of crystals was sufficient to stabilize the foam structure. Interestingly, the linear PP achieved a higher VER than the PP-ethylene copolymer. This suggested that high VER foams can be produced with linear PP despite its low melt strength when the crystalline structures are optimized.

## 3. Experimental Section

### 3.1. Experimental Materials

The plastics used in this study were a linear PP (DM55, Borealis, Vienna, Austria) and a PP-ethylene random co-polymer (SEP550, Honam, Seoul, Korea). The melting temperature (*T_m_*) and crystallization temperature (*T_c_*) of both polymers were measured using DSC analysis (DSC Q2000, TA Instruments, New Castle, DE, USA) under atmospheric pressure. The *T_m_* and *T_c_* of the linear PP are 163 and 117 °C, respectively, while the *T_m_* and *T_c_* of the PP-ethylene copolymer are 146 and 107 °C, respectively. The polymer resins were compression molded to films 0.4 mm in thickness with a hot press at 200 °C. Upon pressure release, the molded films were immediately quenched with a large reservoir of water at 13 °C. Afterwards, the films were cut into circular discs that are approximately 4 and 6.5 mm in diameter for HPDSC analysis and foaming visualization experiments, respectively. The blowing agent used was CO_2_ (99.8% pure, Linde Gas Inc., Munich, Germany).

### 3.2. Measurement of Isothermal Crystallization with High-Pressure Differential Scanning Calorimetry

The crystallization kinetics of each PP was studied with a HPDSC system (DSC 204 HP, NETZSCH, Selb, Bavaria, Germany). Each polymer sample was first heated from 20 to 200 °C and was maintained at 200 °C for 10 min to completely melt the existing crystals. Afterwards, the sample was cooled down to an isothermal temperature (*T_iso_*), and was maintained at *T_iso_* for 60 min. Finally, it was cooled down to 20 °C. The heating/cooling rate for the entire cycle was set constant at 10 °C/min. Figure 4 depicts an example of the temperature cycle used with *T_iso_* = 139 °C. The saturation pressure (*P_sat_*) used in all of the HPDSC analysis was maintained at 6 MPa. The temperature of the sample might differ slightly from the measured temperature, especially during the heating/cooling ramp, since the temperature sensor does not directly measure the sample temperature. However, due to the small sample thickness (0.4 mm), the moderate heating/cooling rate (10 °C), and the short distance of the temperature sensor relative to the sample, the effect of this temperature different was expected to be negligible. A similar conclusion can be made for the visualization system since the location of the temperature sensor (RTD) in the visualization system is also closed to the sample (see Figure 1). Crystallinity measurement was conducted at a range of *T_iso_* for each material to determine the maximum *T_iso_* at which the crystallinity became too low to be measured by the HPDSC. This study determined the suitable range of *T_iso_* for the foaming experiments to investigate the effects of crystals on plastic foaming behavior.

### 3.3. In Situ Observation of Crystal Formation and Foaming Processes

To conduct an experiment, a circular disc shaped plastic sample was placed inside the high temperature/high pressure chamber. A clear polyethylene terephthalate (PET) film (0.127 mm in thickness) with a 1 mm hole punched out in the center was placed beneath each sample, so that the sample was partially unsupported at both the top and bottom surface (see Figure 10). Observation of foaming processes was focused upon that region. This minimized the effects of heterogeneous nucleation and/or formation of cells from pre-existing cavities along the sample-sapphire and sample-PET interfaces, which could be more thermodynamically favorable to cell nucleation within the bulk phase of polymer [64]. This allowed the effects of crystals in semi-crystalline polymers on their foaming behavior to be investigated in an isolated manner. The neighboring regions where the sample was in contact with PET were also captured to demonstrate the differences in foaming behavior in these two different regions. After closing the chamber, a high-pressure blowing agent was injected into the chamber. Meanwhile, the chamber was heated and/or cooled according to a programmed temperature profile, during which the blowing agent was dissolved into the polymer sample. During this annealing process, crystallization could take place depending on the processing temperature, pressure and annealing time, and the high-speed camera took snapshots of the crystal formation *in situ*. Afterward, a gas release valve was triggered to open, thus causing a sudden release of gas pressure. The rapid pressure drop caused a thermodynamic instability within the polymer to initiate the foaming process, which was captured by a high-speed camera *in situ*. By adjusting the resistance of the gas exit path with a metered valve, a specific pressure drop rate was obtained.

## 4. Conclusions

A polymer visualization system with accurate heating/cooling program control was developed successfully to observe crystal formations under high temperature and pressure, which complements HPDSC analysis to achieve in-depth understanding of crystallization and melting behaviors of semi-crystalline polymers under typical foaming conditions. At the same time, this system allows *in situ* observation of the subsequent plastic foaming processes induced by rapid depressurization. Combining these two capabilities, this system allows effective investigation of the interrelationships between crystallization kinetics and cell nucleation, growth and deterioration phenomena in plastic foaming processes, which is an important step to establish thorough understanding in plastic foaming processes of semi-crystalline polymers. Via *in situ* observation of a linear PP and a PP-ethylene copolymer, the capability of the foaming visualization system has been demonstrated. It was shown that bubbles nucleated around crystals at low temperatures, which was due to the exclusion effect of CO_2_ and the tensile stresses induced by bubble growth to the constrained amorphous regions between adjacent crystals. A high crystallinity was needed to generate foams with the maximum VER for the linear PP, and its processing temperature window was very narrow due to its low melt strength. Meanwhile, the processing temperature window for PP-ethylene copolymer was much wider. Nevertheless, this study suggests that a high VER could be achieved with both the linear PP and the PP-ethylene copolymer when the crystalline structures are optimized. Due to the wide operating temperature and pressure window of the visualization system, it can be effectively used for a wide range of polymer crystallization and foaming research in the future.

## Abbreviations

*A*: pre-exponent parameter for the cell nucleation rate equation, #/cm^3^-s
*F*: ratio of a nucleated bubble’s volume to a spherical bubble’s volume with the same radius, dimensionless
*k_b_*: Boltzmann’s Constant, m^2^-kg/s^2^-K
*J*: cell nucleation rate, #/cm^3^-s
*P_bub,cr_*: bubble pressure at the critical radius, Pa
*ΔP_local_*: local system pressure around a bubble, Pa
*P_sat_*: saturation pressure, Pa
*P_sys_*: system pressure, Pa
−*dP_sys_/dt*|_avg_: average pressure drop rate, Pa/s
*R_cr_*: critical radius, cm
*T_c_*: crystallization temperature, °C
*T_iso_*: isothermal temperature, °C
*T_m_*: melting temperature, °C
*T_sys_*: system temperature, °C
*W*: free energy barrier for cell nucleation, J
*θ_c_*: contact angle, °
*γ_lg_*: surface tension at the polymer/gas interface, N/m
